# Survey of childhood empyema in Asia: Implications for detecting the unmeasured burden of culture-negative bacterial disease

**DOI:** 10.1186/1471-2334-8-90

**Published:** 2008-07-11

**Authors:** Batmunkh Nyambat, Paul E Kilgore, Dong Eun Yong, Dang Duc Anh, Chen-Hsun Chiu, Xuzhuang Shen, Luis Jodar, Timothy L Ng, Hans L Bock, William P Hausdorff

**Affiliations:** 1 Division of Translational Research, International Vaccine Institute, Seoul, South Korea; 2 Department of Laboratory Medicine, College of Medicine, Yonsei University, Seoul, South Korea; 3 National Institute of Hygiene and Epidemiology, Hanoi, Vietnam; 4 Chang Gung Children's Hospital and Chang Gung University College of Medicine, Taipei, Taiwan; 5 Beijing Children's Hospital affiliated to Capital Medical University, Beijing, PR China; 6 GlaxoSmithKline Biologicals, Rixensart, Belgium

## Abstract

**Background:**

Parapneumonic empyema continues to be a disease of significant morbidity and mortality among children despite recent advances in medical management. To date, only a limited number of studies have assessed the burden of empyema in Asia.

**Methods:**

We surveyed medical records of four representative large pediatric hospitals in China, Korea, Taiwan and Vietnam using *ICD*-10 diagnostic codes to identify children <16 years of age hospitalized with empyema or pleural effusion from 1995 to 2005. We also accessed microbiology records of cultured empyema and pleural effusion specimens to describe the trends in the epidemiology and microbiology of empyema.

**Results:**

During the study period, we identified 1,379 children diagnosed with empyema or pleural effusion (China, n = 461; Korea, n = 134; Taiwan, n = 119; Vietnam, n = 665). Diagnoses of pleural effusion (n = 1,074) were 3.5 times more common than of empyema (n = 305), although the relative proportions of empyema and pleural effusion noted in hospital records varied widely between the four sites, most likely because of marked differences in coding practices. Although pleural effusions were reported more often than empyema, children with empyema were more likely to have a cultured pathogen. In addition, we found that median age and gender distribution of children with these conditions were similar across the four countries. Among 1,379 empyema and pleural effusion specimens, 401 (29%) were culture positive. *Staphylococcus aureus *(n = 126) was the most common organism isolated, followed by *Streptococcus pneumoniae *(n = 83), *Pseudomonas aeruginosa *(n = 37) and *Klebsiella *(n = 35) and *Acinetobacter *species (n = 34).

**Conclusion:**

The age and gender distribution of empyema and pleural effusion in children in these countries are similar to the US and Western Europe. *S. pneumoniae *was the second leading bacterial cause of empyema and pleural effusion among Asian children. The high proportion of culture-negative specimens among patients with pleural effusion or empyema suggests that culture may not be a sufficiently sensitive diagnostic method to determine etiology in the majority of cases. Future prospective studies in different countries would benefit from standardized case definitions and coding practices for empyema. In addition, more sensitive diagnostic methods would improve detection of pathogens and could result in better prevention, treatment and outcomes of this severe disease.

## Background

Globally, respiratory diseases are a leading cause of morbidity and mortality among both children and adults [1,2]. In developing countries, children less than 5 years of age are at high risk for severe, life-threatening disease associated with bacterial and viral pathogens [3]. *Streptococcus pneumoniae *is a major respiratory pathogen, and the spectrum of clinical presentations highly associated with this pathogen includes bacteremic and non-bacteremic presentations of pneumonia as well as parapneumonic effusions or empyema [4,5].

Several studies from developed countries suggest that the prevalence of empyema and pleural effusion may be increasing [6-10]. In these countries, pediatric empyema is often quickly identified and treated promptly with surgical intervention or pharmacologic therapy [11,12]. Nevertheless, empyema is associated with prolonged hospitalization stays (mean ~7 days) and with a case-fatality rate of about 5–7% [13]. While predictors of empyema in hospitalized children are not well-known, it appears that host factors may play a predisposing role [14]. In developing countries, severe pneumonia in children may be associated with necrotizing changes in a unilateral or bilateral pattern [15,16]. In developing countries, antecedent conditions such as malnutrition, measles or infection with antibiotic-resistant organisms may increase the risk of severe pneumonia accompanied by empyema [17,18]. In South Korea, the 7-valent conjugate pneumococcal vaccine (PCV7) was licensed in 2002 and coverage has reached to ~30% in the infant age group [19]. In Taiwan, PCV7 was introduced in 2006 and coverage is <10%. In both Korea and Taiwan, pneumococcal polysaccharide vaccines (PPV) are available but uptake has been low in older children and adults [20]. In China and Vietnam, the PCV7 has not been licensed (likely to occur in next 2 years) and uptake of PPV has also been slow and coverage is low (~1%).

The mean age of children with empyema and pleural effusion in developed country studies is 3–6 years with 50% to 80% of cases occurring in males [21-23]. In previous studies, bacterial pathogens that included *Staphylococcus aureus*, *S. pneumoniae*, *Streptococcus pyogenes *and *Haemophilus influenzae *type b (Hib) were associated with empyema in children [24,25]. However, an emerging body of literature now suggests that *S. pneumoniae *in particular is a major cause of empyema and that selected serotypes of pneumococcus may play an important role in this emerging disease pattern [10].

In the Asia-Pacific region, a limited number of clinical and laboratory studies suggest that *S. pneumoniae *may also be the most common etiologic agent in empyema and pleural effusion specimens [26-28]. In order to better understand epidemiologic and microbiologic patterns of empyema and pleural effusion among diverse populations of Asian children <16 years of age, we undertook a retrospective review of hospital records in four countries.

## Methods

### Overview

For this study, we selected four tertiary care medical centers specializing in treatment of children: Chang Gung Children's Hospital (Taipei, Taiwan), Beijing Children's Hospital (Beijing, China); Yonsei University Hospital (Seoul, Korea) and the National Pediatric Hospital affiliated with the Ministry of Health (Hanoi, Vietnam). Each provided a representative sample of patients treated at major tertiary care hospitals, maintained patient hospital discharge and laboratory records in computerized databases, and permitted collaboration with experienced clinical researchers. The study protocol was approved by the International Vaccine Institute Institutional Review Board. Local investigators met with hospital clinicians and microbiologists to assess the extent of their experience with complicated pneumonia and routine microbiology laboratory practices for pleural fluid specimen testing. Hospital clinical and microbiology departments were asked to identify and collect information on clinically-diagnosed empyema. Due to variations in hospital record availability, the data collection periods varied somewhat–1995–2004 in China and Korea, 2000–2005 in Taiwan and 1996–2005 in Vietnam.

### Laboratory and medical records data collection

In each hospital, data collection for patients with empyema was restricted to hospitalized patients <16 years of age. Hospital microbiologists accessed laboratory databases and record books to compile a listing of pleural effusion and empyema specimens that were tested by microbiologic culture. Collaborating investigators also reviewed computerized microbiology records for reports of culture-negative and culture-positive empyema or pleural effusion specimens and provided lists of organisms isolated in the specimens. In each study hospital, cultures for anaerobes were not routinely performed. In addition, these hospital laboratories did not routinely culture for fungi, mycobacterium and parasites.

*International Classification of Diseases *(*ICD*-10) codes were used to conduct searches of computerized hospital discharge record databases in each collaborating hospital. Each hospital's medical records department staff created discharge record databases to identify patients discharged with an *ICD*-10 diagnostic code corresponding to pyothorax with fistula (J86.0), pyothorax without fistula (J86.9) or pleural effusion (J90). To identify the total number of hospitalized pneumonia patients, we provided a standardized listing of etiology-specific and non-specific *ICD*-9 or *ICD*-10 diagnostic codes. Following exclusion of confidential identifying information such as national registration number, the medical records discharge databases were transferred to the International Vaccine Institute for review and analysis.

### Data analyses

Where possible for each hospital, demographic and hospitalization characteristics were analyzed to describe epidemiologic patterns by age group, date of hospital discharge, type of specimen collected and organism isolated. However, the data on *S. pneumoniae *in particular from Taiwan did not include information such as age and admission dates. An analysis of specimen collection and patient discharge dates was performed to assess the seasonal distribution of patients with culture-positive and culture-negative specimens. Statistical comparisons were performed to identify significant differences in the distribution of different variables by calculating a 95% confidence interval and a critical ratio (Z) test with *P*-value (significance level *P *< 0.05) for the difference between two independent proportions.

## Results

### Hospitalizations for empyema and pleural effusion

From the four study hospitals, this review identified a total of 1,379 patients diagnosed with empyema or pleural effusion (Table 1): 665 in Vietnam, 461 in China, 134 in Korea and 119 in Taiwan. The number of hospitalizations due to empyema and pleural effusion increased over time, at least up to 2002, with some yearly fluctuations. However, there was no significant change over time in number of hospitalizations due to empyema and pleural effusion among the four study hospitals. Overall in the four countries, 60% of patients with empyema and pleural effusion were male. A preponderance of males was noted in all countries (62% in China and Korea and 57% in Taiwan and Vietnam).

**Table 1:** Distribution of patients with empyema or pleural effusion during hospitalization in four hospitals in China, Korea, Taiwan and Vietnam, 1995–2005.

Time Period	Hospital Location				Total
		
	Vietnam*	China^†^	Korea^†^	Taiwan^‡^	
1995–1996	48 (7%)	76 (16%)	19 (14%)	--	143 (10%)
1997–1998	113 (17)	114 (25)	7 (5)	--	234 (17)
1999–2000	146 (22)	71 (15)	31 (23)	18 (15)	266 (19)
2001–2002	179 (27)	83 (18)	35 (26)	51 (43)	348 (25)
2003–2005	179 (27)	117 (25)	42 (31)	50 (42)	388 (28)

Total	665 (100)	461 (100)	134 (100)	119 (100)	1,379 (100)

*Vietnam hospital data were available for 10 years (1996–2005).

^†^China and Korea hospital data were available for 10 years (1995–2004).

^‡^Taiwan hospital data were only available for 6 years (2000–2005).

Among the 1,379 patients, 305 (22%) were diagnosed with empyema and 1,074 (78%) with pleural effusion (Table 2). In China, all patients were recorded as having pleural effusion. In Vietnam and Korea, children with pleural effusion outnumbered those classified with empyema; 4 to 1 in Vietnam and 2 to 1 in Korea. Hospitalization data from Taiwan showed the lowest frequency of children with pleural effusion – only 4%, while 96% were coded as empyema. To put our results for empyema and pleural effusion in context, we identified the total number of hospitalizations for pneumonia among children <15 years of age in each hospital. The Vietnam study hospital had the most pneumonia hospitalizations (n = 54,673) followed by 14,770, 12,254 and 11,193 in the China, Taiwan and Korea study hospitals, respectively. Among these pneumonia hospitalizations, empyema and pleural effusion were most commonly identified in China (3.1%) followed by Vietnam and Korea (1.2% each) and Taiwan (1.0%).

**Table 2:** Age distribution of hospitalized children with empyema and pleural effusion in China, Korea, Taiwan and Vietnam.

Age group	Vietnam N (%)		China N (%)		Korea N (%)		Taiwan N (%)		Total N (%)
		
	Empyema	Pleural effusion	Empyema	Pleural effusion	Empyema	Pleural effusion	Empyema	Pleural effusion	
1	50 (35.7)	140 (26.7)	--	56 (12.1)	6 (11.8)	26 (31.3)	13 (11.4)	1 (20.0)	292 (21.2)
2	31 (22.1)	64 (12.2)	--	25 (5.4)	16 (31.4)	17 (20.5)	8 (7.0)	1 (20.0)	162 (11.7)
3	15 (10.7)	32 (6.1)	--	21 (4.6)	7 (13.7)	12 (14.5)	19 (16.7)	0 (0)	106 (7.7)
4	14 (10.0)	30 (5.7)	--	23 (5.0)	1 (2.0)	4 (4.8)	20 (17.5)	1 (20.0)	93 (6.7)
5	5 (3.6)	28 (5.3)	--	31 (6.7)	2 (3.9)	5 (6.0)	24 (21.1)	0 (0)	95 (6.9)
6	2 (1.4)	12 (2.3)	--	36 (7.8)	5 (9.8)	0 (0)	11 (9.6)	0 (0)	66 (4.8)
7	2 (1.4)	21 (4.0)	--	41 (8.9)	0 (0)	1 (1.2)	9 (7.9)	0 (0)	74 (5.4)
8	3 (2.1)	18 (3.4)	--	48 (10.4)	3 (5.9)	0 (0)	3 (2.6)	0 (0)	75 (5.4)
9	2 (1.4)	25 (4.8)	--	35 (7.6)	1 (2.0)	5 (6.0)	0 (0)	1 (20.0)	69 (5.0)
10	4 (2.9)	36 (6.9)	--	35 (7.6)	2 (3.9)	2 (2.4)	1 (0.9)	0 (0)	80 (5.8)
11	2 (1.4)	20 (3.8)	--	22 (4.8)	0 (0)	0 (0)	1 (0.9)	0 (0)	45 (3.3)
12	6 (4.3)	34 (6.5)	--	24 (5.2)	2 (3.9)	1 (1.2)	1 (0.9)	0 (0)	68 (4.9)
13	1 (0.7)	27 (5.1)	--	36 (7.8)	1 (2.0)	0 (0)	2 (1.8)	1 (20.0)	68 (4.9)
14	1 (0.7)	22 (4.2)	--	23 (5.0)	3 (5.9)	6 (7.2)	0 (00)	0 (0)	55 (4.0)
15	2 (1.4)	16 (3.0)	--	5 (1.1)	2 (3.9)	4 (4.8)	2 (1.8)	0 (0)	31 (2.2)

Total	140 (100)	525 (100)	--	461 (100)	51 (100)	83 (100)	114 (100)	5 (100)	1,379 (100)

NOTE. Data for China and Korea are for 1995–2004; for Taiwan, 2000–2005; and Vietnam, 1996–2005.

NOTE: Specimen type in China was not stratified by appearance.

Overall, 21% of all patients diagnosed with empyema or pleural effusion in the four study hospitals were less than 1 year of age (Table 2). The Vietnam study hospital had the highest proportion of patients (29%) with empyema and pleural effusion in that age group followed by Korea (24%) and China and Taiwan (12% each). Children with pleural effusions in Korea and Vietnam were significantly younger than in China (*P *< .0001). Notably, although the relative proportion of patients classified with empyema or pleural effusion was markedly different in China and Taiwan, nonetheless the proportion of patients (12%) less than 1 year of age was identical. The mean age of children with empyema and pleural effusion in China was 7.6 years compared with 5.1, 4.1 and 3.2 years in Vietnam, Taiwan and Korea, respectively.

**Figure 1:**
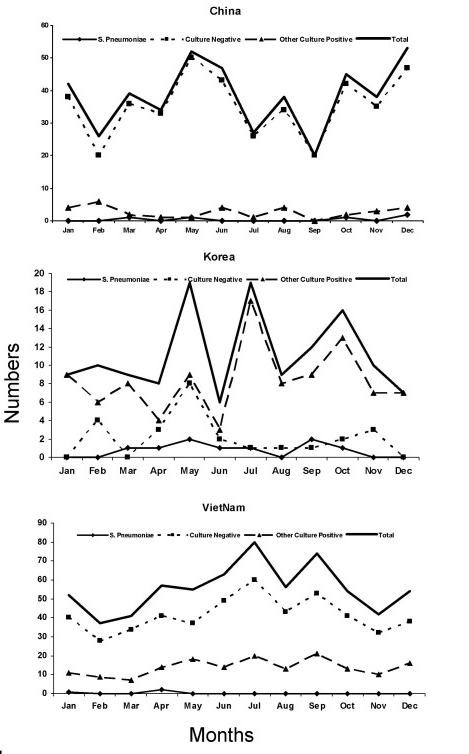
**Monthly distribution of microbiologic culture results from testing of empyema and pleural fluid specimens in China, Korea and Vietnam**.* *China and Korea (1995 to 2004); Taiwan (2000 to 2005) and Vietnam (1996 to 2005).

There was no obvious seasonality in the occurrence of pleural effusion and empyema or in pneumococcal empyema in these four countries. Overall, children with empyema and pleural effusion were more likely to be hospitalized in China during the months of May, June and December (Figure 1), while in Korea, more hospitalizations occurred during May, July and October. In Vietnam, June and September were the most common months for hospitalizations for empyema and pleural effusion.

### Culture-positive and culture-negative pleural fluid and empyema specimens

A total of 1,379 empyema (n = 310) and pleural effusion (n = 1,069) specimens were tested by bacterial culture, and 71% (n = 980) were negative for any bacterial organism (Table 3). Ninety-two percent of Chinese specimens tested negative compared with 75% that were culture-negative in Vietnam, 28% in Taiwan and 19% in Korea. Among the 310 empyema specimens, 61% (n = 188) were culture-positive compared with 20% (n = 211) of pleural effusion specimens that were culture-positive (*P *< 0.05). If the 211 children with culture-positive pleural effusion specimens are grouped into the empyema category, the percentage of culture-positive specimens increased from 61% to 72% (*P *< 0.05).

**Table 3:** Organisms identified in empyema and pleural effusion specimens from China, Korea, Taiwan and Vietnam, 1995–2005.

Cultured Organism	Empyema N (%)	Pleural effusion N (%)	Total N (%)
Culture negative	122 (39)	858 (80)	980 (71)
Gram negative	51 (17)	104 (10)	155 (11)
Gram positive			
*S. pneumoniae*	68 (22)	15 (1)	83 (6)
Others	69 (22)	92 (9)	161 (12)

Total	310 (100)	1,069 (100)	1,379 (100)

*S. aureus *was the most common organism isolated in Korea and Vietnam (29% and 48%, of all positive bacterial isolates, respectively), while *S. pneumoniae *predominated in Taiwan (77% of all positive bacterial isolates) (Table 4). No single pathogen dominated among the few culture-positive samples from China. *Acinetobacter *and *Pseudomonas *organisms was the 2^nd ^and 3^rd ^most common pathogens reported in Korea (28% and 11% of all positive bacterial isolates, respectively), followed by *S. pneumoniae *(8% of all positive bacterial isolates). In Vietnam, *Klebsiella *and *Pseudomonas *species were isolated in 17% and 11% of all positive bacterial isolates. *S. pneumoniae *was isolated from 83 patients including 66 (80%) in Taiwan, 9 (11%) in Korea, 5 (6%) in China and 3 (3%) in Vietnam. Of the 83 pneumococcal isolates, 82% (n = 68) were from empyema cases, mostly from Taiwan, compared to 18% (n = 15) from pleural effusion cases.

**Table 4:** Distribution of isolates in empyema and pleural effusion specimens by country.

Empyema or Pleural Fluid Isolates	Vietnam	China	Korea	Taiwan	Total
	(n = 665)	(n = 461)	(n = 134)	(n = 119)	(n = 1,379)
Culture negative	496 (74.6)	424 (92.0)	25 (18.7)	33 (27.7)	978 (70.9)
*Staphylococcus aureus*	81 (12.2)	7 (1.5)	32 (23.9)	6 (5.0)	126 (9.1)
*Streptococcus pneumoniae*	3 (0.5)	5 (1.1)	9 (6.7)	66 (55.5)	83 (6.0)
*Pseudomonas aeruginosa*	19 (2.9)	5 (1.1)	12 (9.0)	1 (0.8)	37 (2.7)
*Klebsiella spp*.	28 (4.2)	4 (0.9)	3 (2.2)	0 (0)	35 (2.5)
*Acinetobacter spp*.	4 (0.6)	0 (0)	30 (22.4)	0 (0)	34 (2.5)
*Escherichia coli*	9 (1.4)	1 (0.2)	2 (1.5)	1 (0.8)	13 (0.9)
*Enterococcus spp*.	2 (0.3)	1 (0.2)	8 (6.0)	0 (0)	11 (0.8)
*Haemophilus influenzae *type b	9 (1.4)	0 (0)	1 (0.7)	4 (3.4)	14 (1.0)
*Enterobacter spp*.	1 (0.2)	2 (0.4)	6 (4.5)	0 (0)	9 (0.7)
*Streptococcus spp*.	8 (1.2)	0 (0)	1 (0.7)	1 (0.8)	10 (0.7)
*Stenotrophomonas maltophilia*	0 (0)	1 (0.2)	3 (2.2)	0 (0)	4 (0.3)
*Mycoplasma pneumoniae*	0 (0)	0 (0)	0 (0)	4 (3.4)	4 (0.3)
*Corynebacterium *spp.	0 (0)	2 (0.4)	0 (0)	0 (0)	2 (0.2)
Candida albicans	2 (0.3)	0 (0)	1 (0.7)	0 (0)	3 (0.2)
*Citrobacter spp*.	2 (0.3)	0 (0)	0 (0)	0 (0)	2 (0.2)
Fungi	0 (0)	2 (0.5)	0 (0)	0 (0)	2 (0.2)
*Micrococcus spp*.	0 (0)	2 (0.4)	0 (0)	0 (0)	2 (0.2)
*Burkholderia cepacia*	0 (0)	0 (0)	1 (0.7)	0 (0)	1 (0.1)
*Haemophilus parainfluenzae*	0 (0)	1 (0.2)	0 (0)	0 (0)	1 (0.1)
*Anaerobic*	1 (0.2)	0 (0)	0 (0)	0 (0)	1 (0.1)
*Neisseria spp*.	0 (0)	1 (0.2)	0 (0)	0 (0)	1 (0.1)
*Staphylococcus epidermidis*	0 (0)	1 (.02)	0 (0)	0 (0)	1 (0.1)
*Staphylococcus intermedius*	0 (0)	1 (0.2)	0 (0)	0 (0)	1 (0.1)
viridans group streptococci	0 (0)	1 (0.2)	0 (0)	0 (0)	1 (0.1)
*Veillonella spp*.	0 (0)	0 (0)	0 (0)	1 (0.8)	1 (0.1)
*Clostridium spp*.	0 (0)	0 (0)	0 (0)	1 (0.8)	1 (0.1)
*Prevotella spp*.	0 (0)	0 (0)	0 (0)	1 (0.8)	1 (0.1)

Total	665 (100)	461 (100)	134 (100)	119 (100)	1,379 (100)

## Discussion

Our study results show that pleural effusion and empyema occur in Asia among young children at rates similar to those observed elsewhere (1–3% of pneumonia admissions) [29-31]. However, the clinical differentiation between pleural effusion and empyema may be interpreted differently from one country to another, as suggested by the great difference in the proportion of the two types reported in the four countries studied. For example, in China, 100% of the pleural effusion and empyema cases were coded only as pleural effusion, but it is likely that these included many empyema cases. In contrast, in Taiwan, only 4% of the cases were coded as pleural effusion, suggesting either that virtually no pleural fluid samples were taken from children diagnosed with uncomplicated pleural effusions or that all patients from whom samples are taken are automatically coded as "empyema". In Korea and Vietnam, the proportions were more mixed, but coding for pleural effusion nonetheless predominated. Regardless of the distinction, these findings emphasize the importance of looking at coding for both pleural effusion and empyema in order to understand the epidemiology of complicated pneumonias.

In this review, one-third of the empyema patients identified were less than 2 years of age confirming that empyema may be more likely to occur in young children than in older children. In India, one-third of hospitalized children with empyema were <5 years of age [26]. The mean age (4~5 years) of children with empyema and pleural effusion in our study was similar to findings elsewhere [32-34].

The absence of a distinct seasonality in the distribution of empyema patients in this study suggests weather may not be an important contributing factor or that the etiologic agents of empyema and pleural effusions specimens could be a heterogeneous group of infectious agents. This conclusion is supported, in part, by data from microbiologic cultures of empyema and pleural fluid showing a wide variety of Gram-positive and Gram-negative organisms as well as fungi and culture-negative specimens.

Our study identified several children whose empyema or pleural fluid cultures grew bacterial pathogens normally associated with community-acquired lower respiratory tract disease including Hib, *S. pneumoniae *and *S. aureus *[35-37]. These findings are consistent with a growing number of reports suggesting that much childhood empyema could be vaccine-preventable [38,39]. In our study hospitals, data on bacterial species also suggest either that a large proportion of children may acquire Gram-negative pathogens as nosocomial infections or that a number of bacterial isolates are contaminants of laboratory cultures [40,41]. Previous reviews or case series describing bacterial organisms isolated from children with empyema suggest that Gram-positive as well as Gram-negative organisms may invade the pleural space [42,43].

In our study, a high proportion (71%) of all empyema and pleural fluid specimens grew no bacterial pathogen. This finding is consistent with a number of previous studies suggesting that the negative cultures are not the result of limitations in routine microbiology laboratory procedures. The negative cultures more likely are due to the widespread use of antibiotics (including inappropriately chosen or dosed antibiotics) as well the potential for severe viral lower respiratory tract disease to be associated with pleural effusion or bacterial superinfections resulting in necrotizing pneumonia and empyema [44,45]. This study collected data from existing computerized hospital discharge databases and laboratory records but individual patient medical records were not accessed to obtain information on prior treatment with antibiotics. In general, in our previous studies, we have found that parent- or patient-reported prior use of antibiotics is often not recorded in medical records. In addition, the high rate of negative cultures may be due to the presence of fastidious organisms such as anaerobic bacteria [46]. Based on our previous studies in Asia, we have found that many hospital laboratories do not use anaerobic culture media. According to a recent study reported by Song JH et al, the prevalence of penicillin resistance in *S. pneumoniae *isolates was 71.4% in Vietnam followed by Korea (54.8%), Taiwan (38.6%) and China (23.4%) [47]. Given the high proportion of bacterial culture-negative pleural fluid specimens, a more complete assessment of parapneumonic pleural effusions or empyema in prospective studies could apply non-culture-based antigen detection or polymerase chain reaction tests to detect both bacterial and viral pathogens. These tests have been used to identify children with culture-negative pneumococcal infections [43,48-50]. These sensitive diagnostic tools can help us to better understand the burden of disease, trends over time, epidemiological differences among countries, patient demography, symptomatology, etiological agents and rational treatment. Unfortunately, at present, these laboratory techniques are only generally available in research laboratories.

A number of investigators have shown that laboratory testing of pleural fluid or empyema specimens in children with pneumonia can provide important insights into the origins of the pneumonia [51,52]. In Asia relatively few children hospitalized with serious pneumonia undergo thoracocentesis diagnostic procedures to identify pathogens in pleural or empyema fluid, which are relatively less culturally acceptable in Asian populations [53]. Nevertheless, given the number of children identified in our retrospective survey, prospective multi-center studies in Asia are likely to yield substantial numbers of patients with empyema or complicated pleural effusions and shed light on the etiological agent associated with parapneumonic empyema and pleural effusions.

In this study, we found that children with empyema were significantly more likely to have positive bacterial cultures compared with children in whom pleural effusion specimens were collected. These data are consistent with previous studies suggesting that empyema fluid is the result of established infections and inflammatory reactions [28,54]. However, given the fact that clinicians in Asia have also found it necessary to collect clinical specimens from patients with pleural effusions, it is likely that prospective studies that include an evaluation of bacterial pathogens in pleural effusions specimens will yield a more accurate picture of the total burden of disease associated with invasive bacterial pneumonia and parapneumonic bacterial infections.

This study has some limitations. First, as a retrospective review, our data collection, analysis and reporting were restricted to that available in hospital databases or logbooks. Thus, for some years of data, incomplete patient information precluded further analysis. Computerized hospital administrative databases were accessed by collaborating study investigators. Nevertheless, in some hospitals we found that current levels of data entry limited the amount of clinical and historical data available. The increasing use of electronic medical records in Asia suggests that additional patient clinical and laboratory data are likely to be available in a number of countries in coming years. Finally, because the hospitals in our review did not record more than one *ICD*-10 diagnostic code, we were unable to determine other clinical conditions that the children may have had at the time of their hospitalization and thus we could not determine the proportion of children with pneumonia or lower respiratory tract infections among those who had empyema or pleural fluid specimens collected.

Similarly, as the hospital laboratories did only limited testing, there was no means to identify underlying causes of lower respiratory tract disease in infants and young children. In addition, most clinical laboratories in the study countries do not routinely preserve bacterial isolates from pleural fluid specimens because they lack resources and awareness of the utility of such specimens for research and advancement of treatment.

## Conclusion

Future incidence studies in such hospitals to determine the true burden of parapneumonic empyema may be feasible if catchment areas for study sites can be well-defined. Recent studies from France [42,55] suggest that the incidence of empyema may vary over time. Our results suggest that prospective surveillance for pneumonia with empyema or pleural effusions could be established as part of larger surveillance for severe bacterial infections including meningitis and sepsis. Surveillance for invasive bacterial diseases can be improved by: a) applying standardized case definitions; b) create, disseminate and implement standard pediatric guidelines for treatment of pneumonia, empyema and other syndromes associated with invasive bacterial diseases; c) requiring report of clinical laboratory specimens from normally sterile sites that are culture-positive for Hib, *S. pneumoniae*, *N. meningitidis *and other invasive bacterial pathogens; and d) implementing standard operating procedures that maximize capacity for detection of invasive bacterial pathogens in hospital laboratories. Future prospective studies of empyema will benefit from standardized case definitions and coding practices for empyema as well as pleural effusion.

## Competing interests

The authors declare that they have no competing interests.

## Authors' contributions

BN and PEK conceived and designed the study, assisted with data collection, performed the data analyses and drafted the study manuscript. DEY, DDA, C–HC and XS implemented standardized methods for hospital data collection, verified data sources and accuracy and participated in writing of the study manuscript. LJ, TLN, HLB and WPH provided input into data collection, reviewed outputs from data analysis and assisted in editing of the study manuscript.

## Pre-publication history

The pre-publication history for this paper can be accessed here:


